# Comparative Evaluation of Anesthetic Efficacy of 4% Articaine and 2% Lignocaine as Inferior Alveolar Nerve Block in Patients With Symptomatic Irreversible Pulpitis in Permanent Mandibular First Molars: A Double-Blinded Randomized Clinical Trial

**DOI:** 10.7759/cureus.89090

**Published:** 2025-07-30

**Authors:** Suchismita Chakraborty, Mayakiru Pakyntein, Rubi Kataki, Adrija Deka, Debosmita Roy, Diganta Borah

**Affiliations:** 1 Conservative Dentistry and Endodontics, Regional Dental College, Guwahati, IND

**Keywords:** articaine, epinephrine, inferior alveolar nerve, lignocaine, local anesthesia, mandibular first molar, pain management, root canal

## Abstract

Introduction: Achieving effective anesthesia during root canal treatment is particularly challenging in cases of symptomatic irreversible pulpitis due to the inflamed pulpal environment, which compromises anesthetic efficacy. Articaine and lignocaine are commonly used local anesthetics in endodontics, differing in onset time, duration, and pain control. The objective of this study was to compare the anesthetic efficacy of 2% lignocaine combined with 1:80,000 epinephrine with 4% articaine combined with 1:100,000 epinephrine during root canal treatment of permanent mandibular first molars in patients with symptomatic irreversible pulpitis.

Method: 100 patients, 18-30 years old, with symptomatic irreversible pulpitis in permanent mandibular first molars without any periapical pathology reported to the Department of Conservative Dentistry and Endodontics, Regional Dental College, Guwahati. Following the provision of written informed consent, participants were randomly assigned to receive a conventional inferior alveolar nerve block using either 1.8 mL of 2% lignocaine with 1:80,000 epinephrine (n = 50) or 1.7 mL of 4% articaine with 1:100,000 epinephrine (n=50). Randomization was performed by an individual who did not take part in data acquisition. To avoid identification, the independent observer covered 50 cartridges containing 2% lignocaine and 50 cartridges containing 4% articaine with blue and pink colored tapes, respectively. Patients were subjectively assessed for lip anesthesia. Absence/presence of pulpal anesthesia was tested by using electric pulp stimulation, and absence/presence of pain was assessed by using a Short-Form McGill Pain Questionnaire (Appendix). Data were analyzed using IBM Corp. Released 2011. IBM SPSS Statistics for Windows, Version 20.0. Armonk, NY: IBM Corp., with the Student’s paired t-test to compare outcomes between the groups.

Results: The articaine group showed a faster onset of anesthesia (4.3 min vs. 7.8 min), longer duration (120.8 ± 3 min vs. 64.9 ± 5.2 min), and better pain control during root canal treatment (mean pain score: 1.09 vs. 1.63) compared to the lignocaine group. These differences were statistically highly significant (p < 0.001).

Conclusion: Articaine demonstrated faster onset, longer duration, and better pain control than lignocaine, making it a preferred choice for inferior alveolar nerve block in permanent mandibular first molars with symptomatic irreversible pulpitis.

## Introduction

Effective pain management is crucial in minimizing patient discomfort and lowering anxiety and fear while receiving dental treatments. It is frequently difficult to provide deep anesthesia during root canal treatment, particularly for teeth having symptomatic irreversible pulpitis. Hyperalgesia, prostaglandin-induced peripheral nociceptors' sensitization, psychological variables, acute tachyphylaxis, blood flow, and inflammatory reactions that alter the pH of local tissue are some of the plausible factors [1].

Local anesthetics are essential for preventing or reducing pain, as they block nerve impulses at the site of administration. The effectiveness of articaine and lidocaine, two common local anesthetics in dentistry, might vary depending on their onset, duration of action, and ability to reduce discomfort during root canal therapy. Despite their structural differences, articaine and lignocaine belong to a similar class of anesthetics, having the amide group. Articaine has both amides and an ester ring, but lignocaine lacks an ester ring [2].

Due to the presence of an ester ring, articaine undergoes biotransformation both in the liver, by hepatic microsomal enzymes, and in the plasma through hydrolysis by plasma esterases. Elimination of articaine occurs via the kidneys. It has been shown that between 5 and 10% are eliminated unaltered. The ester group in articaine is important for protein binding in addition to its function in metabolism. Many factors affect the onset and duration. Articaine has a faster onset of action compared to lignocaine. This is partly due to its higher lipid solubility and better diffusion through soft and hard tissues. However, the duration of anesthesia varies depending on whether it affects the pulp or the soft tissue. Additionally, the duration can differ based on the technique used, such as IANB versus infiltration [2,3].

Although several studies [1-5] have compared the efficacy of lignocaine versus articaine, this study is the first to compare the two using this specific concentration of anesthetic with epinephrine-2% lignocaine with 1:80k epinephrine and 4% articaine with 1:100,000 epinephrine in root canal treatment procedures in patients diagnosed with symptomatic irreversible pulpitis in the permanent mandibular first molar.

The purpose of the study was to assess the effectiveness of 2% lignocaine with 1:80,000 epinephrine and 4% articaine with 1:100,000 epinephrine as inferior alveolar nerve blocks in individuals with symptomatic irreversible pulpitis in permanent mandibular first molars undergoing root canal treatment procedures.

According to the null hypothesis, there is no discernible difference between 4% articaine, 1:100,000 epinephrine, and 2% lignocaine, 1:80,000 epinephrine, in terms of anesthetic efficacy.

## Materials and methods

Study design and setting

This four-month prospective comparison study was carried out at the Regional Dental College in Guwahati. The Institutional Ethics Committee of Regional Dental College provided ethical approval numbered No. RDC/29/2011/Pt.-I/1742.

Inclusion Criteria

Using the formula n = ([Za/2 × s]/E), where Za/2 = 1.96 (at 95% confidence level), is the estimated population standard deviation, and E is the desired error of the estimated population mean, 100 cases of symptomatic irreversible pulpitis in permanent mandibular first molars of medically healthy ASAI having the ability to understand and sign the consent, aged 18-30 years (to ensure uniformity in biological response and minimize confounding variables), were taken for the study. Diagnosis was established using a combination of subjective and objective criteria. Subjective symptoms included spontaneous pain, an extended reaction to cold stimulation (lasting more than 30 seconds), and discomfort exacerbated by postural changes. Objective tests, such as the cold test using Endo-Frost (Coltene-Roeko, Germany) and a positive result on the electric pulp test (Waldent, India), are performed to confirm pulpal inflammation. Radiographic examination revealed deep carious lesions approaching the pulp, with no signs of periapical pathology. Only teeth with a confirmed diagnosis of irreversible pulpitis, based on American Association of Endodontists guidelines, were included to ensure homogeneity and accuracy in evaluating anesthetic efficacy.

To participate in the study, each individual was required to have at least one neighboring tooth and a clinically sound contralateral canine. Alternatively, a contralateral canine was considered acceptable if it exhibited no indicators of deep dental caries, significant restorations, periodontal complications, history of trauma, or sensitivity. This contralateral canine, which was not subjected to anesthesia, functioned as a control to confirm both the proper operation of diagnostic equipment and the patient’s responsiveness. All pre- and post-injection assessments were conducted by trained personnel blinded to the anesthetic volume administered. A probe coated with conductive gel was placed on the middle third of the buccal surface of the test tooth. The electric current was gradually increased over 25 seconds, from 0 to 80 mA, and the current level at which the patient first perceived sensation was recorded. Pain perception was assessed using the Short Form McGill Pain Questionnaire (SF-MPQ) (Appendix). Only individuals reporting moderate to severe pain were included in the final sample.

Exclusion Criteria

Patients who are allergic to anesthetic solution, have a history of paresthesia or nerve injury, take medications that affect pain perception, are diabetic, are hypertensive, are pregnant, are smokers, are alcoholics, or have other comorbidities are excluded from the study. Patients were assessed radiographically; permanent mandibular first molars with periapical radiolucency and any abnormalities like open apices and extra roots were excluded.

Protocol

Patients were assigned at random to receive either of the anesthetics. Randomization was performed by an individual who did not take part in data acquisition. To avoid identification, the independent observer covered 50 cartridges containing 2% lignocaine and 50 cartridges containing 4% articaine with blue and pink colored tape, respectively. Employees in charge of distributing the covered cartridges provided the codes for randomization; they were not involved in the drug delivery or outcome analysis. Since neither the patient nor the operator was aware of the specifics of the kind of local anesthesia administered, the experiment's double-blind feature was guaranteed.

Procedure

**Figure 1:**
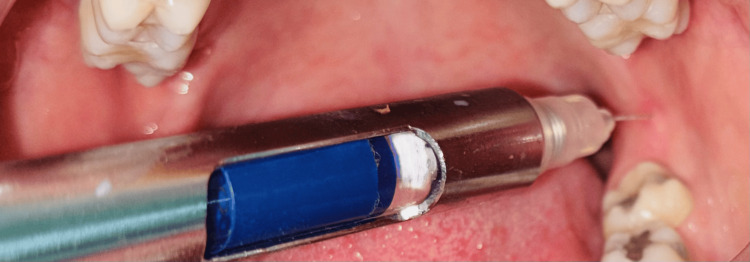
Inferior alveolar nerve block administered using lignocaine

**Figure 2:**
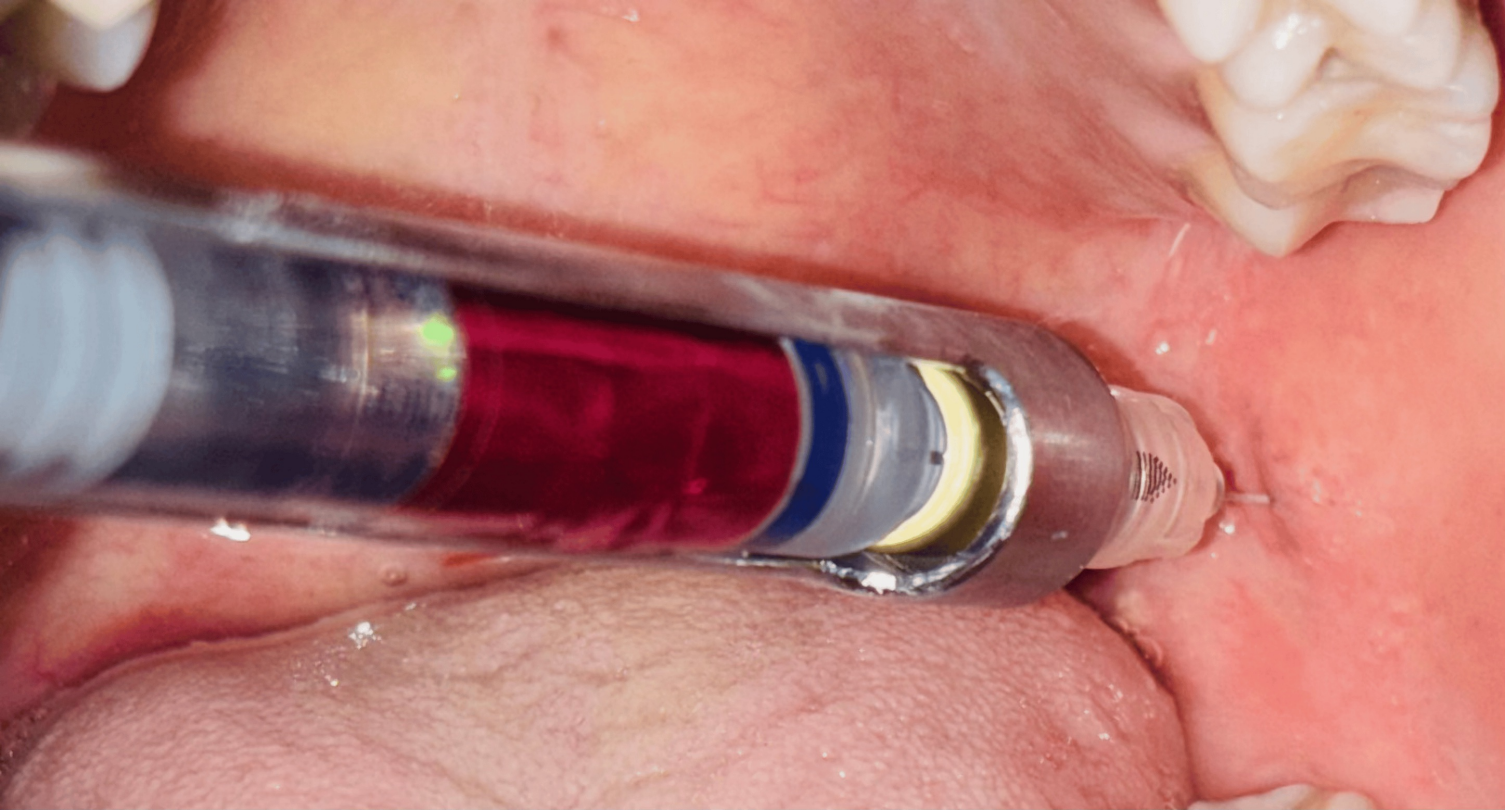
Inferior alveolar nerve block administered using articaine

Before the start of the procedure, the patient provided written informed consent. The study groups were divided as follows: Group I (n=50): 2% lignocaine, 1:80,000 epinephrine, and Group II (n=50): 4% articaine, 1:100,000 epinephrine. A 20% benzocaine local anesthetic was administered topically at the site of injection. One minute later, an inferior alveolar nerve block injection was administered. After the needle was inserted and made contact with the bone, it was withdrawn 1-2 mm. In group 1, 2% lignocaine anesthetic agent taped in blue was administered (Figure 1), and in group 2, 4% articaine taped in pink was delivered (Figure 2). Each patient was asked subjectively and objectively to confirm the action of local anesthesia. To assess pain after caries removal and access cavity preparation, an SF-MPQ has been used. Parameters that were analyzed in this study are the rate of onset, duration, and pain sensation during the root canal treatment.

Outcome Measures

The onset of action was determined based on the time at which pulpal anesthesia was objectively confirmed. Using the electric pulp tester, the duration of action was calculated in minutes based on subjective and objective values. Pain perception was assessed using a standardized SF-MPQ.

Data Collection and Analysis

A Microsoft Excel (Redmond, USA) spreadsheet from 2007 was used to compile and enter recorded data. To find out the differences in outcome parameters between the two groups, IBM Corp. Released 2011. IBM SPSS Statistics for Windows, Version 20.0. Armonk, NY: IBM Corp. was used to administer the student's paired t-test. P-values below 0.05 were regarded as statistically significant.

## Results

The study participants were distributed based on age and gender as shown in Table 1. Amongst 100 participants, 60 were male, 40 were female; 60 patients were in the age range of 18-25 years, and 40 patients were in the age range of 25-30 years. The study groups were divided into Group 1, as lignocaine, and Group 2, as articaine. The parameters assessed were onset, duration, and pain perception while carrying out root canal treatment.

**Table 1:** Demographic variables of the study participants

Gender	18-25 yrs	25-30yrs	Total
Male	35	25	60
Female	25	15	40
Total	60	40	100

Table 2 shows that Group 2 had a faster onset of action, with a mean score of 4.3 minutes as compared to Group 1 with a mean score of 7.8 minutes. Also, the average length of action of Group 1 was 64.9 minutes, whereas Group 2 exhibited a greater duration of action, 120.8 minutes. The short-form McGill pain questionnaire was used to assess pain perception after caries removal and access cavity preparation, and the results indicated that Group 2 (1.09) had a lower mean pain score than Group 1 (1.63). Therefore, Group 2 demonstrated a more rapid onset, prolonged anesthetic action, and an effective reduction in pain compared to Group 1; a statistically significant difference was noted (p<.001). 

**Table 2:** Comparison of outcome parameters between the two study groups Group 1: lignocaine anesthesia, Group 2: articaine anesthesia, *statistically significant - paired t-test (p<0.001*)

Parameters	Groups	Number	Mean	Standard deviation	P value
Onset of action	1	50	7.8	0.8	0.001*
	2	50	4.3	0.4	
Duration of action	1	50	64.9	3.0	0.001*
	2	50	120.8	5.2	
Pain perception	1	50	1.63	0.640	0.001*
	2	50	1.09	0.258	

## Discussion

The present study focused on the comparison of the duration of action, onset, and effectiveness in pain reduction between lignocaine and articaine for root canal treatment procedures. The use of 2% lidocaine with 1:80,000 epinephrine, as opposed to the 1:100,000 formulation, is a strategic choice to optimize anesthetic efficacy. A higher concentration of epinephrine intensifies vasoconstriction at the injection site, limiting systemic absorption and prolonging the duration of nerve blockade. While both concentrations are considered safe in healthy individuals, the 1:80,000 ratio was preferred to enhance clinical outcomes without compromising patient safety.

To assess the standardized onset and duration of pulpal anesthesia, an electric pulp test is utilized in this study, similar to other studies [6,7]. Based on the findings of Dreven et al. [8], the criteria for pulpal anesthesia were developed. The non-responsive patient with a reading of 80 indicates severe pulpal anesthesia. Certosimo et al. [9] found that teeth that were generally asymptomatic had discomfort when the electric pulp tester reading was less than 80. Haas et al. [10] emphasized that the gold standard for confirming pulpal anesthesia is a reading of 80 on the electric pulp tester, which is widely accepted in clinical and research settings.

The results of this study demonstrated that articaine provided significantly superior pain control compared to lignocaine, as evidenced by lower pain scores and improved patient comfort during procedures. The SF-MPQ was selected as the primary tool for evaluating pain due to its ability to effectively capture both sensory and affective dimensions of pain in a time-efficient manner. Unlike the full McGill Pain Questionnaire, which can be burdensome to administer, the SF-MPQ has proven valuable in providing quantitative intensity ratings as well as qualitative descriptions of pain. Previous research, including work by Melzack [11] and Edirisinghe et al. [12], supports its reliability and validity in clinical settings. For these reasons, the SF-MPQ was used to assess pain control after caries removal and access cavity preparation in this study, allowing for a more precise comparison between the anesthetic efficacy of articaine and lignocaine.

Hassan et al. [13] reported that articaine exhibited a significantly more rapid onset of anesthesia compared to lignocaine, attributing this to its superior lipid solubility and enhanced tissue permeability, which facilitate faster diffusion across nerve membranes. Similarly, Kakroudi et al. [14] observed that articaine not only produced a faster onset but also a longer duration of anesthesia, making it especially beneficial in endodontic procedures where prolonged anesthesia is desirable.

According to the current study, lignocaine 2% with 1:80,000 epinephrine can produce pulpal anesthesia in 64.9 minutes. Bhargava et al. [15] emphasized that because the aromatic ring is replaced, articaine has a 1.5-fold higher potency than lignocaine. The duration and effectiveness of a local anesthetic are strongly influenced by its degree of protein binding, which governs how long the drug remains active at the site of action. This correlation has been supported by the findings of Vishal et al. [16] and Bansal et al. [17], who reported that anesthetic agents with higher protein-binding affinity tend to produce more prolonged and reliable anesthesia. Furthermore, studies conducted by Potocnik et al. [18] and Borchard et al. [19] demonstrated that articaine exhibits superior efficacy compared to lignocaine in blocking A-delta fibers, responsible for sharp, localized pain, thereby contributing to its more rapid onset and deeper anesthetic effect. These findings collectively support the clinical preference for articaine in procedures requiring effective pulpal anesthesia, especially in challenging cases.

Structurally, articaine is unique among local anesthetics due to its hybrid nature, possessing both an amide and an ester group within its thiophene ring structure [20]. This dual characteristic contributes to its increased lipophilicity, enhanced diffusion through soft and hard tissues, and rapid hydrolysis by plasma esterases, allowing for safer systemic metabolism and a lower risk of toxicity [21-23]. The presence of the thiophene ring, as opposed to the benzene ring in lignocaine, further improves its lipid solubility, facilitating a quicker onset and deeper nerve block [24].

Kanaa et al. [25] demonstrated that 4% articaine with epinephrine produced superior buccal infiltration anesthesia in mandibular molars compared to 2% lignocaine, which is often considered less effective in such dense bony areas. Similar findings were reported by Robertson et al. [26], who found that articaine was more effective in achieving pulpal anesthesia for permanent mandibular first molars in patients with symptomatic irreversible pulpitis, a clinical condition often resistant to traditional anesthetic approaches.

Overall, the combination of rapid onset, deep and sustained anesthesia, efficient tissue penetration, and favorable safety profile, if proper anesthetic technique is followed, positions articaine as a clinically advantageous local anesthetic in modern endodontics.

The future scope of our research includes increasing the sample size to improve statistical validity, incorporating patient-specific factors like age, gender, health status, and tooth type, and expanding to various endodontic procedures such as retreatments and regenerative cases. This will support a more personalized and comprehensive understanding of anesthetic efficacy.

Study limitations

A smaller sample size could affect the results' generalizability, even if our study strategy, which involves blocking the inferior alveolar nerve in mandibular molars during root canal therapy operations, accounts for inter-individual variability. This study focused on the mandibular first molar to ensure consistency. However, due to anatomical and physiological differences, the results may not be directly applicable to maxillary teeth, where anesthetic efficacy could vary. Furthermore, not enough research was done on variables that can affect how pain is perceived, such as patient anxiety and pain threshold.

## Conclusions

Compared to lignocaine, articaine showed a faster onset, prolonged anesthetic effect, and less pain perception. For patients receiving root canal therapy, the prolonged duration of action makes the procedure more comfortable and painless. Using articaine during root canal therapy may help patients feel more satisfied and experience less anxiety throughout the procedure. The results of the study indicate that for root canal treatment procedures, articaine with epinephrine is a more effective local anesthetic option than lignocaine.

## Figures and Tables

**Figure 3:**
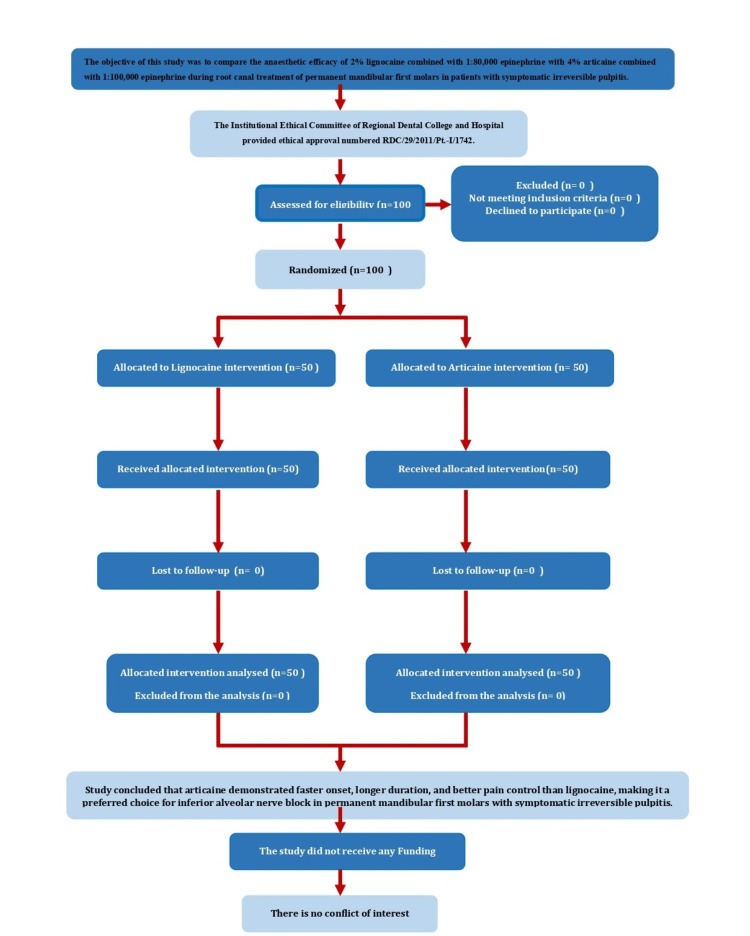
PRIRATE 2020 flowchart

**Figure 4:**
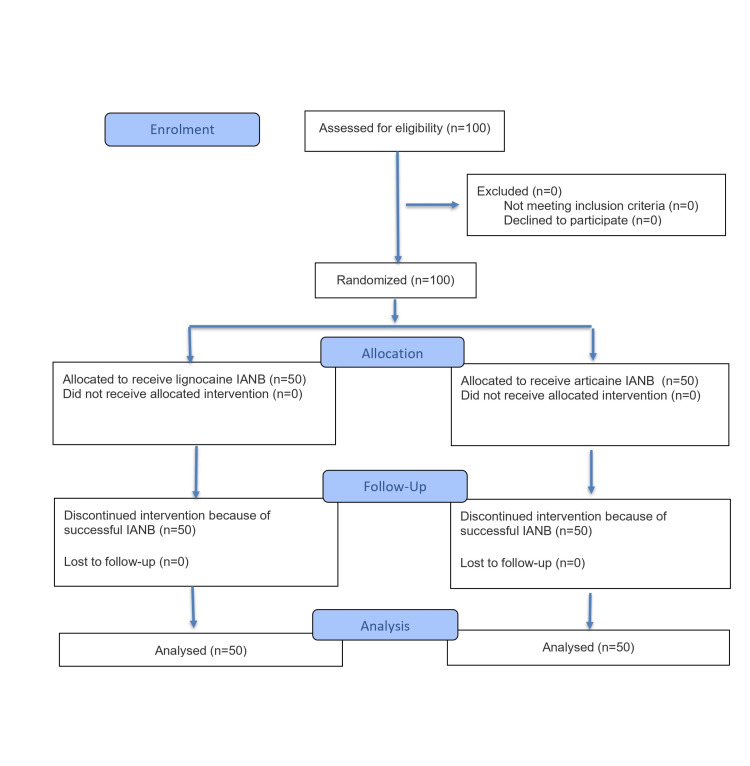
CONSORT 2025 Flowchart IANB: inferior alveolar nerve block

**Figure 5:**
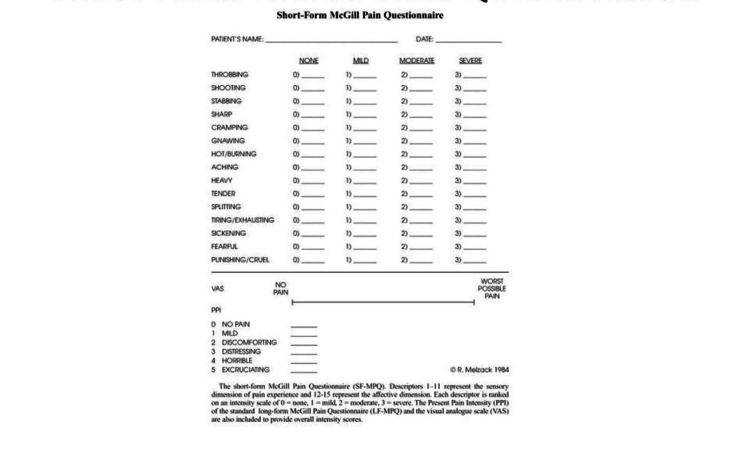
Short-Form McGill Pain Questionnaire

**Figure 6:**
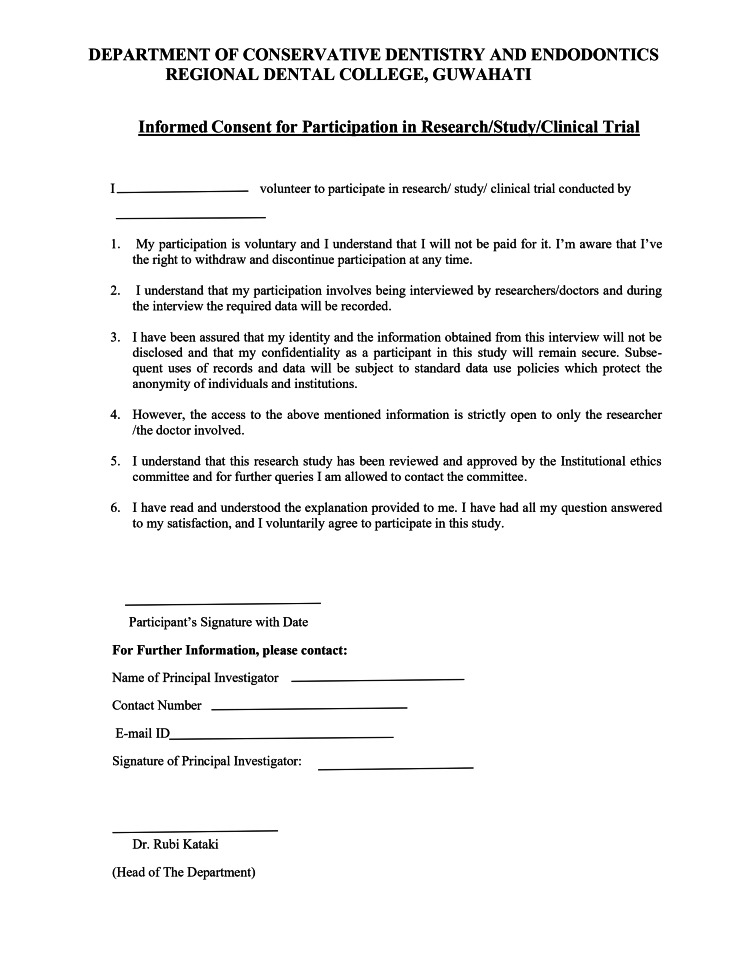
Written informed consent form for participation in study

**Figure 7:**
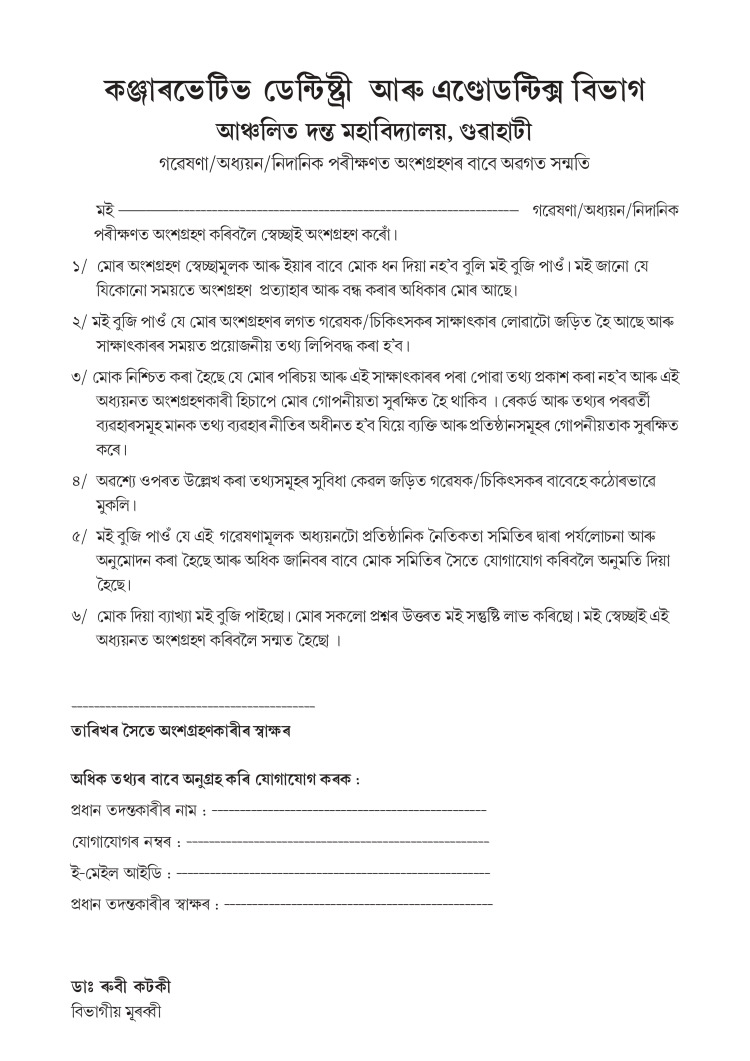
Written informed consent form for participation in study in local language
